# Digital Screening for Early Identification of Cognitive Impairment: A Narrative Review

**DOI:** 10.1002/wcs.70009

**Published:** 2025-07-05

**Authors:** Ester Cornacchia, Aurora Bonvino, Giorgia Francesca Scaramuzzi, Daphne Gasparre, Roberta Simeoli, Davide Marocco, Paolo Taurisano

**Affiliations:** ^1^ Department of Translational Biomedicine and Neuroscience – DiBraiN University of Bari Aldo Moro Bari Italy; ^2^ Department of Humanistic Studies University of Naples Federico II Naples Italy

**Keywords:** cognitive screening, diagnostic accuracy, digital tools, neurocognitive disorders

## Abstract

As longevity increases, cognitive decline in older adults has become a growing concern. Consequently, an increasing interest in the potential of digital tools (e.g., serious games (SG) and virtual reality (VR)) for early screening of Mild Cognitive Impairment (MCI) is emerging. Traditional cognitive assessments like the Mini‐Mental State Examination (MMSE) and Montreal Cognitive Assessment (MoCA) are widely used but have limitations related to cultural bias and manual scoring, while their digital adaptations, such as MOCA‐CC, maintain diagnostic accuracy while offering remote administration and automated scoring. Innovative tools, such as the Virtual Super Market (VSM) test and Panoramix Suite, instead, assess cognitive domains like memory, attention, and executive function while promoting engagement and preserving ecological validity, making assessments more reflective of real‐world tasks. Several studies show that these tools exhibit strong diagnostic performance, with sensitivity and specificity often exceeding 80%. However, although digital tools offer advantages in accessibility and user engagement, challenges remain concerning technological literacy, data privacy, and long‐term validation. Future research should focus on validating these tools across diverse populations and exploring hybrid models that combine traditional and digital assessments, as digital tools show promise in transforming cognitive screening and enabling earlier interventions for cognitive decline.

This article is categorized under:

Psychology > Development and Aging
Neuroscience > Cognition

## Graphical abstract

As longevity increases, digital tools like VR and serious games transform cognitive screening, offering accuracy, engagement, and real‐world relevance. Overcoming challenges in technological literacy, privacy, and validation is essential to unlock their full potential and enable earlier interventions for cognitive decline.

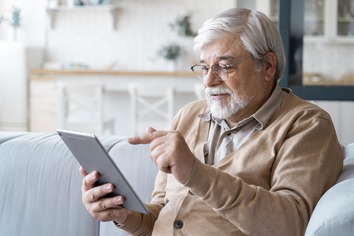

## 1 Introduction

As modern society experiences an increase in longevity, there is also a rise in the prevalence of gradual and long‐term cognitive decline, linked both to healthy and pathological aging. Hence, due to the considerable impact on health and social care planning and delivery, the challenge for clinicians is to develop affordable and practical methods for screening older adults, making early‐stage treatment more effective by identifying those at risk of developing dementia before its onset (Livingston et al. 2020). Dementia consists of a collection of cognitive deficits, including memory impairment and agnosia, aphasia, apraxia, or impaired executive functioning, affecting daily activities (Holsinger et al. 2007). On the other hand, Mild Cognitive Impairment (MCI), marking a prodromal stage, offers a critical window of opportunity for interventions, potentially delaying the progression of dementia (Petersen 2009). However, it is frequently misdiagnosed in the primary care setting due to multiple interrelated factors that inhibit the accurate assessment of cognitive performance, such as comorbidities, reduced expertise of practitioners, limitations of assessment tools, and inadequate integration of cognitive assessments in electronic health records, restricting continuative monitoring (Sabbagh, Boada, Borson, et al. 2020). Cognitive screening is the initial phase in evaluating impairment, also useful for early detection of other issues (Taghavi et al. 2024). The Mini‐Mental State Examination (MMSE) (Folstein et al. 1975) is widely used for the screening of dementia. Nevertheless, there are additional tests for dementia screening in healthcare settings, available at no cost and with comparable diagnostic efficacy, like the Mini‐Cog and the Addenbrooke's Cognitive Examination–Revised, or the Montreal Cognitive Assessment (MoCA), with the highest accuracy for MCI detection (Tsoi et al. 2015). However, traditional cognitive assessment tools have several limitations, including biases related to culture, gender, and education, as well as issues with long test intervals and learning effects.

In the last decades, numerous studies have explored the potential of using technology and Artificial Intelligence to support neuropsychological diagnostic processes from neurodevelopment to neurodegeneration (Battista et al. 2017; Cerasuolo et al. 2024; Luongo et al. 2024; Simeoli et al. 2024). The recent advances in technology have led to the development of serious games (SG), addressing these challenges and potentially replacing conventional cognitive screening methods (Taghavi et al. 2024), through games designed with purposes beyond mere entertainment and from which individuals with cognitive impairments may benefit. These games can assess different cognitive variables while maintaining engagement and stimulating cognitive activity during the daily life, contributing to slowing or reducing further cognitive deterioration (Tong et al. 2017). Furthermore, remote smartphone‐based tracking allows individuals to monitor their cognitive health over time, enhancing their commitment to well‐being (Pillemer et al. 2016) while ensuring ecological validity and reducing the burden of in‐clinic visits for participants in clinical trials (Schlemmer and Desrichard 2018), thereby fostering broader participation. Significant advances in sensor technology, virtual reality (VR), and SG have made digital neuropsychological tools and mobile health applications (mHealth apps) increasingly relevant and widely adopted for diagnosing NCDs (McCarthy et al. 2024). Despite the promise of smartphone‐based cognitive assessments, feasibility issues, including openness of elders to participate, compliance, attrition, and privacy concerns, represent relevant challenges. In addition, validity, alignment with gold‐standard tests, and reliability, including the variability in hardware, operating systems, and uncontrolled test environments, are major concerns (Öhman et al. 2021). The development of digital tools for cognitive evaluation is still in its early stages, and many of the available tests have yet to be validated in large and controlled studies, constraining their use by both concerned individuals and physicians (Sabbagh, Boada, and Borson 2020). Even though smartphone‐based cognitive assessments are promising for collecting larger datasets, their validity, compared to traditional neuropsychological tests, is underexplored (Butler et al. 2024; De Anda‐Duran et al. 2024).

Additionally, the implementation and accessibility of digital cognitive assessment tools must be considered in light of the gender digital divide. For example, recent data show that more than 61% of women aged 55–74 in the EU do not use Internet banking, reflecting a broader trend of lower digital engagement among older women compared to men (Eurostat 2023). These disparities may affect familiarity and openness to use digital tools, limiting the potential of interventions addressing early cognitive decline. Therefore, gender representation and digital literacy differences should be considered in both the design and use of these technologies to ensure inclusivity and reduce inequalities.

This review synthesizes studies on innovative digital tools, like VR and SG, to assess cognitive impairment. The main goal is to explore how these perform compared to conventional assessments in identifying early cognitive decline, and their effectiveness, usability, and overall utility. To achieve this, the present review seeks to discuss the following topics: (i) What evidence exists regarding the accuracy of tools employed for early cognitive impairment screening? (ii) What are the key characteristics of these tools? Are they based on traditional tests or innovative paradigms like gamification or VR? (iii) Which cognitive domains are assessed? How do these digital tools correlate with traditional tests? (iv) Are digital assessments perceived by users?

## 2 Material and Methods

Thirty relevant research articles were identified and summarized in the table below (Table 1). Each study explored various digital interventions, from traditional neuropsychological tests digitized and SG to mobile applications and VR, and measured their performance accuracy to detect MCI early.

**TABLE 1:** Study characteristics, cognitive domains, correlation, diagnostic accuracy parameters, participant satisfaction of digital tools reviewed. This table provides sample characteristics, cognitive domains assessed, correlation with traditional neuropsychological tests, diagnostic accuracy parameters, participant satisfaction in various digital tools to early detection of mild cognitive impairment (MCI).

Study (year, country)	Sample size (controls/MCI)	Mean age (controls/MCI)	Cognitive domains	Correlation	Diagnostic accuracy parameters	Participant satisfaction
Memória et al. (2014, Brazil)	76 (41/35)	71.7/73.8	Memory, language, attention, executive function	High correlation with MoCA (*r* = 0.76, *p* < 0.001)	AUC = 0.8, sensitivity = 81%, specificity = 73%; no gold standard comparison	NR
Yu et al. (2015, China)	118 (55/63)	72/74	Attention, executive function, memory, language	Good correlation with MoCA (*r* = 0.93, *p* < 0.001)	AUC = 0.97, sensitivity = 96%, specificity = 87%; no gold standard comparison	NR
Zygouris et al. (2015, Greece)	55 (21/34)	66.6/70.3	Memory, attention, executive function, spatial navigation, visuospatial abilities	Moderate correlation with MMSE and duration (*r* = −0.209, *p* < 0.05)	sensitivity = 82%, specificity = 95%; no significant improvement over MMSE alone	NR
Curiel et al. (2016, USA)	98 (64/34)	74/78	Semantic memory	Correlation not reported (NR)	AUC = 0.93, sensitivity = 85%, specificity = 84%; no gold standard comparison	NR
Scharre et al. (2017, Germany)	45 (21/24)	75/—	Orientation, language, memory, executive function	Comparisons with MMSE showed significance (*p* = 0.0452)	AUC = 0.78, sensitivity = 62%, specificity = 81%; comparison with MMSE found similar sensitivity	NR
Müller et al. (2017, Germany)	50 (20/30)	67/65	Attention, auditory comprehension, verbal working memory, numerical knowledge, visual memory, visuospatial abilities, executive function	Time‐in‐air significantly longer in aMCI compared to HC (*p* = 0.003)	AUC = 0.88, sensitivity = 81%, specificity = 72%; significant difference compared to cCDT sensitivity = 62.5%, specificity = 83.3%	NR
Zygouris et al. (2017, Greece)	12 (6/6)	63/64.5	Memory, attention, executive function, spatial navigation, visuospatial abilities	Positive correlations with MMSE and MoCA; moderate correlation with Trail Making Test (TMT‐B)	Sensitivity = 94%, specificity = 89%; correlation aligned with MMSE and MoCA scores	NR
Valladares‐Rodriguez et al. (2018, Spain)	11 (8/3)	68.3/75	Memory, attention, executive function, visuospatial ability	Episodic memory correlated with Rey Auditory Verbal Learning Test (RAVLT) (*r* = 0.68, *p* < 0.01)	Sensitivity = 100%, specificity = 100% (combined model)	93.4% found games entertaining
Ichii et al. (2020, Japan)	224 (121/103)	77/81	Attention, memory, executive function, spatial cognition	Significant correlation with FUCAS (*r* = −0.617, *p* = 0.040)	Sensitivity = 78%, specificity = 54%; no other gold standard comparison	NR
van der Hoek et al. (2019, Netherlands)	82 (45/37)	82.6/84.7	Memory, processing speed	Correlation with MoCA (MTX speed: *r* = 0.39, *p* < 0.000)	AUC = 0.85, sensitivity = 85%, specificity = 80%; no gold standard comparison	NR
Robens et al. (2019, Germany)	131 (67/64)	65.9/67.9	Visuospatial abilities, executive function, motor skills, attention, memory	Good correlation with MMSE (*r* = 0.44, *p* < 0.001)	Sensitivity = 83%, specificity = 56%; NR	NR
Eraslan Boz et al. (2020, Turkey)	210 (52/37)	67.56/70.41	Memory, attention, executive function, spatial navigation	Moderate correlations with MMSE and other verbal memory tests	AUC not reported; sensitivity = 85%, specificity = 74%; VSM + MMSE = 92%, best combination sensitivity = 100%	NR
Alegret et al. (2020, Spain)	276 (154/122)	68 (naMCI 66/aMCI 68)	Memory	Significant correlations with Aβ42 and tau proteins	Sensitivity = 73%, specificity = 72%	65.2% rated excellent
Fung and Lam (2018, Hong Kong)	606 (509/97)	69/73	Episodic memory, attention, visuospatial ability	Moderate correlation with CMMSE (*r* = 0.53, *p* < 0.001)	AUC = 0.793; sensitivity = 86%; specificity = 75%; NR	NR
Rodriguez‐Salgado et al. (2021, Cuba)	99 (53/46)	70/72	Associative memory, processing speed, executive function	Strong associations with other verbal and visual memory tasks	AUC = 0.94; sensitivity = 87%; specificity = 85%; MoCA performance lower at AUC = 0.	
Yan et al. (2021, China)	126 (64/62)	74/82	Learning and memory, executive functions, language, attention	Moderate positive correlation with MMSE (*r* = 0.429, *p* < 0.001) and strong correlation with MoCA (*r* = 0.645, *p* < 0.001)	AUC = 0.87; sensitivity = 85%; specificity = 79%	NR
Karapapas and Goumopoulos (2021, Greece)	10 (4/6)	75.25/76.67	Attention, visual‐motor perception, short‐term memory, executive functions	Correlations with traditional assessments not reported	Sensitivity = 88%, specificity = 82%; NR	NR
Konstantinidis et al. (2021, Greece)	180 (90/90)	68/71	Reaction time, attention, memory, visuomotor coordination	Moderate positive correlations with MMSE (*r* = 0.505) and MoCA (*r* = 0.463)	Sensitivity = 84%, specificity = 63%	NR
Ye et al. (2022, China)	57 (35/22)	67/72	Memory, processing speed, executive function, attention	Strong correlation with MoCA (*r* = 0.77)	AUC = 0.84; sensitivity = 85%; specificity = 83%	97.3% satisfaction rate
Paterson et al. (2022, Australia)	91 (40/51)	69/73	Associative memory, processing speed, executive function	Correlations with MMSE (*r* = 0.61) and Stroop interference (*r* = 0.48)	AUC = 0.67; sensitivity = 65%; specificity = 70%	NR
Cheah et al. (2022, Singapore)	118 (59/59)	63/68	Memory, executive function	High correlation with ROCF (*r* = 0.988)	AUC = 0.91; sensitivity = 85%; specificity = 90%; cost‐effective compared to traditional methods	NR
Valladares‐Rodríguez et al. (2022, Spain)	30 (10/14)	75.62/81.24	Episodic memory, semantic memory, procedural memory	Correlations not reported	Sensitivity = 100%, specificity = 100%; no other gold standard comparison	Average rating 4.55/5 for HC, 4.13/5 for MCI
Oliva and Losa (2022, Italy)	114 (70/44)	75.5/78.1	Orientation, language, memory, attention, executive function	High correlation with MMSE (*r* = 0.735)	AUC = 0.726–0.889; sensitivity = 83%; specificity = 50%	NR
Zhang et al. (2023, China)	220 (198/22)	69.83/72.47	Executive function, cognitive flexibility	High correlation with TMT traditional scores	AUC = 0.726; sensitivity = 73%; specificity = 75%	NR
Klil‐Drori et al. (2024, Canada)	88 (42/46)	70/18	Memory, executive function, processing time	Strong correlation with MoCA (*p* < 0.005)	AUC = 0.84; sensitivity = 91%; specificity = 90%	NR
Zhang et al. (2024, China)	64 (26/38)	64.6/67.5	Visuospatial, memory	Significant correlation with ROCF (*r* = 0.75)	AUC = 0.852; sensitivity = 78.95%; specificity = 84.62%	NR
Ambrosini et al. (2024, Italy/Spain)	89 (45/44)	76.5/82.8	Speech, attention, memory, cognitive function decline	NR	Sensitivity = 78%, specificity = 73%; NR	NR
Berron et al. (2024, Germany/USA)	199 (97/43)	68.2/69.2	Episodic memory	High correlation with PACC5 (*r* = 0.62)	AUC = 0.83; sensitivity = 82%; specificity = 72%	High, with low distraction reported
McMurray et al. (2024, USA)	147 (115/115)	67/71	Abstract reasoning, constructive ability, prioritizing, numerical problem‐solving, visual–spatial ability	Good correlation with ADAS‐Cog	AUC not reported; sensitivity = 63.25%; specificity = 74.07%	NR
Zhao et al. (2022, China)	308 (209/99)	69.8/74.1	Memory, orientation, executive function	NR	AUC = 0.95; sensitivity = 90%; specificity = 92%	High completion rate (97.5%)

To be considered for inclusion in this review, studies had to meet the following criteria:

1. focus on any digital solution for cognitive impairment detection (excluding digital solutions used for monitoring patients with an existing diagnosis of cognitive impairment);
2. include a study sample of adults (>18 years);
3. exclude participants with any acute neurological conditions or major neurocognitive disorders, or institutionalized;
4. include studies focusing on diagnostic accuracy, comparing healthy controls (HC) with MCI only, excluding those diagnosed with dementia;
5. be written in English.

Two investigators (AB and EC) independently extracted data from studies. The variables can be grouped into four key categories: Demographic Information; Tools used for assessment; Cognitive Domains tested; and Performance metrics like sensitivity and specificity. Supporting Information includes correlation with neuropsychological tests for validity and reliability, as well as participant satisfaction to capture user experience.

## 3 Results

### 3.1 Studies Characteristics

The total sample size across the studies reviewed includes 3840 participants. Approximately 53% of the participants were individuals with MCI, while the remaining 47% were HC. The average age of participants across the studies ranged from the late 60s to early 80s. Geographically, these studies spanned multiple regions, including Europe, Asia, North America, and South America, highlighting the global interest in developing and validating digital tools for cognitive assessment. Concerning the diversity of tools, the studies used a range of methods, from VR environments (e.g., the VSM) (Eraslan Boz et al. 2020; Zygouris et al. 2015, 2017), to more traditional neuropsychological tests digitized (e.g., MOCA‐CC and dCDT) (Müller et al. 2017; Yu et al. 2015). These tools covered multiple cognitive domains, including memory, attention, executive function, language, and visuospatial abilities. Accuracy was frequently assessed, with most tools showing strong correlations with standard clinical assessments like the MoCA and MMSE. Sensitivity and specificity varied across studies, with some tools (e.g., FACEmemory and Panoramix Suite) reporting high diagnostic accuracy for detecting MCI. Usability was also a key consideration, with a few tools, particularly those that use VR or gamified platforms, demonstrating high user engagement and satisfaction (Table 1).

### 3.2 Tools Characteristics

The studies included in this review report findings regarding two main categories of cognitive screening tools: traditional tests adapted for digital platforms and innovative tools that employ advanced technology like VR, SG, and speech analysis.

A significant number of the studies included are focused on digital adaptations of traditional neuropsychological assessments, designed to preserve the structural integrity and diagnostic efficacy of classical gold standard diagnostic tools, while integrating technological advancements with the aim of improving precision, efficiency, and scalability. These enhanced tools, such as the computerized versions of the Montreal Cognitive Assessment (MOCA‐CC) (Yu et al. 2015) and BrainCheck (Ye et al. 2022), offer the benefits of automated scoring, precise timing, and remote administration. MOCA‐CC, for instance, provides immediate cognitive performance results by automatically calculating scores, facilitating rapid clinical decision‐making.

The Digital Clock Drawing Test (dCDT) (Müller et al. 2017) is a digital adaptation of a tool that digitizes a widely used traditional test. By enabling participants to draw a clock on a digital device, the test captures intricate details about drawing patterns, stroke sequences, and placement of numbers, offering insights into executive function and visuospatial abilities. The automated scoring system in dCDT increases accuracy and reduces the time required for analysis while providing clinicians with detailed feedback on cognitive performance.

Similarly, tools like the Virtual Super Market (VSM) test (Eraslan Boz et al. 2020; Zygouris et al. 2015, 2017) and Panoramix Suite (Valladares‐Rodriguez et al. 2018; Valladares‐Rodríguez et al. 2022) incorporate more immersive and dynamic approaches through VR and SG. The VSM leverages VR environments to simulate real‐world tasks, like grocery shopping, to assess domains like memory, executive function, and attention. By engaging participants in ecologically valid settings, the VSM provides a more comprehensive view of their cognitive functioning, which can be particularly valuable for detecting early cognitive decline. The Panoramix Suite (Valladares‐Rodriguez et al. 2018; Valladares‐Rodríguez et al. 2022), which includes SG like Episodix, uses gamified tasks to assess episodic, semantic, and procedural memory as well as executive function. These tools promote participant engagement and long‐term adherence, with over 90% of participants rating the games as enjoyable, as a consequence of their interactive and enjoyable nature.

Automated speech analysis tools, such as IA Voce (Ambrosini et al. 2024), represent another category of innovative tools that assess cognitive function through noninvasive means. IA Voce employs a suite of sophisticated algorithms to analyze speech features, including voice periodicity, syllabic structure, and shimmer, to detect subtle changes in cognitive performance (Table 2).

**TABLE 2:** Summary of digital tools for cognitive screening—procedure and participant task. This table provides a description of procedure and participant task used in various digital tools for early detection of mild cognitive impairment (MCI).

Study	Tool name	Detailed tool description
Memória et al. 2014	CANS‐MCI‐Innovative	The Computerized ANalysis System for Mild Cognitive Impairment (CANS‐MCI) is a computer‐based neuropsychological screening tool used to assess memory, language, attention, and executive function. Tasks include word recall, object naming, and solving logic puzzles. Outputs include latency in picture naming, Stroop test latency, and accuracy in matching tasks. It provides automatic comparisons with normative data for cognitive impairment evaluation.
Yu et al. 2015	MOCA‐CC‐Traditional test based	The Computerized Montreal Cognitive Assessment—Chinese Version (MOCA‐CC) is a digital version of the MoCA test used to assess attention, executive function, memory, and language. Participants complete tasks like word recall, clock drawing, and math problems. Outputs include total MoCA scores and subdomain‐specific scores. This tool is primarily used to detect early signs of mild cognitive impairment (MCI).
Zygouris et al. 2015	Virtual Super Market (VSM)‐Innovative	The Virtual Super Market (VSM) is a VR‐based cognitive assessment tool where participants navigate a simulated supermarket. They follow a shopping list, locate specific items, and recall their locations. The tool evaluates memory, attention, executive function, spatial navigation, and visuospatial abilities. Outputs include task duration, number of correct items, and accuracy in locating products.
Curiel et al. 2016	MITSI‐L‐Innovative	The Miami Test of Semantic Interference and Learning (MITSI‐L) is a smartphone‐based cognitive task that evaluates semantic memory. Participants categorize words into semantic groups (e.g., living vs. non‐living). Outputs include the number of correct responses across multiple trials.
Scharre et al. 2017	eSAGE‐Traditional test based	The Electronic Self‐Administered Gerocognitive Exam (eSAGE) is an app‐based cognitive assessment tool used to evaluate orientation, language, memory, and executive function. Participants solve word puzzles, recall lists of words, and answer orientation questions (e.g., date, time). Outputs include total scores across various domains. The tool provides a digital, self‐administered alternative to traditional cognitive exams.
Müller et al. 2017	Digital Clock Drawing Test (dCDT)‐Traditional test based	The Digital Clock Drawing Test (dCDT) is a digital assessment tool designed to assess visuospatial abilities, attention, and executive function. Participants draw a clock by placing numbers and setting the time on a digital device. The system records the process, including time transitions between strokes, total drawing time, and patterns.
Zygouris et al. 2017	Virtual Super Market (VSM)‐Innovative	The Virtual Super Market (VSM) is a VR‐based cognitive assessment tool where participants navigate a simulated supermarket. They follow a shopping list, locate specific items, and recall their locations. The tool evaluates memory, attention, executive function, spatial navigation, and visuospatial abilities. Outputs include task duration, number of correct items, and accuracy in locating products.
Valladares‐Rodriguez et al. 2018	Panoramix Suit‐Innovative	The Panoramix Suite is a series of SG‐based cognitive assessment tools that measure episodic memory, procedural memory, and semantic memory. The suite includes games like Episodix (episodic memory recall), Procedurix (procedural memory tasks), and Semantix (semantic memory tasks).
Ichii et al. 2020	CogEvo‐Innovative	The CogEvo is a PC‐based cognitive assessment tool that evaluates attention, memory, executive function, and spatial cognition. Participants complete tasks such as reacting to flashing lights, recalling lists of items, and solving spatial puzzles. Outputs include reaction times and task accuracy, providing an analysis of cognitive performance across these domains.
van der Hoek et al. 2019	MemTrax Test‐Innovative	The MemTrax Test is a web‐based cognitive monitoring tool designed to assess memory retention and processing speed. Participants view a series of images and must decide whether the current image matches a previously seen one. Outputs include memory performance scores based on speed and accuracy.
Robens et al. 2019	Digital Tree Drawing Test (dTDT)‐Traditional test based	The Digital Tree Drawing Test (dTDT) is an app‐based cognitive test used to evaluate visuospatial abilities, executive function, motor skills, attention, and memory. Participants draw a tree on a digital device, and the system records the drawing time, painting time, and number of colors used.
Eraslan Boz et al. 2020	Virtual Super Market (VSM)‐Innovative	The Virtual Super Market (VSM) is a VR‐based cognitive assessment tool designed to evaluate memory, attention, executive function, and spatial navigation. Participants navigate a virtual supermarket, complete shopping tasks, and recall item locations. Outputs include task duration and accuracy in completing the shopping tasks. The tool focuses on practical cognitive abilities within a simulated real‐world environment.
Alegret et al. 2020	FACEmemory‐Innovative	The FACEmemory is an app‐based cognitive assessment tool that focuses on face recognition memory. Participants are shown a series of faces and must recognize and recall them after a delay. The tool also correlates memory performance with AD‐related biomarkers, such as Aβ42 and tau proteins. Outputs include memory scores related to cognitive performance, and it is used to detect early cognitive decline, particularly related to AD.
Fung and Lam 2018	HK‐VMT‐Innovative	The Hong Kong Virtual Maze Test (HK‐VMT) is a virtual reality‐based cognitive assessment tool that assesses episodic memory, attention, and visuospatial abilities. Participants navigate through a virtual maze, solving spatial navigation problems and recalling visual stimuli. Outputs include episodic memory performance scores, measuring how well participants can remember spatial and visual details of the maze. The tool simulates real‐world navigation challenges.
Rodríguez‐Salgado et al. 2021	BHA Cuban Version‐Traditional test based	The Brain Health Assessment (BHA) Cuban Version is an online cognitive health assessment tool designed to assess associative memory, processing speed, and executive function. Participants complete tasks such as memory recall and problem‐solving. Outputs include associative memory and processing speed scores. The tool is culturally adapted for Cuban participants and is used to detect early signs of cognitive impairment.
Yan et al. 2021	Virtual Super Market Program‐innovative	The Virtual Super Market Program is a VR‐based cognitive assessment tool that measures learning, memory, executive functions, language, and attention. Participants navigate a virtual supermarket, complete shopping tasks, and are assessed on their ability to recall item locations and complete purchases. Outputs include total score and subscores, evaluating performance across multiple cognitive domains. The tool is designed for early detection of cognitive impairments.
Karapapas and Goumopoulos 2021	COGNIPLAT‐Innovative	The COGNIPLAT is a serious game‐based cognitive training and assessment platform designed to assess attention, visual‐motor perception, short‐term memory, and executive functions. Participants complete tasks such as object manipulation, problem‐solving, and memory challenges within a gamified environment. Outputs include task completion time and accuracy, providing insights into cognitive performance across multiple domains.
Konstantinidis et al. 2021	FitForAll (FFA)‐Innovative	The FitForAll (FFA) is a fitness and cognitive assessment platform that integrates physical and cognitive tasks to evaluate reaction time, attention, memory, and visuomotor coordination. Participants engage in both physical activities and cognitive tests such as reaction time and memory recall challenges. Outputs include metrics from both physical and cognitive performance, providing a comprehensive overview of overall cognitive and physical health.
Ye et al. 2022	BrainCheck Battery‐Innovative	The BrainCheck Battery is an app‐based cognitive assessment tool that includes a variety of tests to measure memory, processing speed, executive function, and attention. Participants complete tasks such as word recall, reaction time challenges, and executive function tests like puzzles. Outputs include both overall scores and task‐specific scores, providing a detailed view of cognitive function across multiple domains.
Paterson et al. 2022	BHA‐Traditional test based	The Brain Health Assessment (BHA) is an app‐based cognitive assessment tool used to evaluate working memory, attention, and cognitive flexibility. Participants complete tasks assessing short‐term memory recall, attention, and the ability to switch between tasks. Outputs include cognitive health reports based on performance in various cognitive tasks, providing an overview of the participant's cognitive abilities.
Cheah et al. 2022	Digital Screening System‐Traditional test based	The Digital Screening System is a PC‐based cognitive assessment tool that includes tasks adapted from well‐known neuropsychological tests like the Rey‐Osterrieth Complex Figure. Participants complete tasks assessing memory, visual–spatial ability, attention, and executive function. Outputs include memory performance scores, providing a comprehensive digital alternative for cognitive screening and early detection of cognitive impairments.
Valladares‐Rodríguez et al. 2022	Panoramix 2.0‐Innovative	The Panoramix 2.0 is a SG‐based cognitive assessment tool designed to assess episodic memory, semantic memory, and procedural memory. Participants engage in gamified tasks that test their ability to recall past events (episodic memory), understand and use general knowledge (semantic memory), and perform sequences of actions (procedural memory). Outputs include domain‐specific memory scores, and the tool provides an engaging format for cognitive testing.
Oliva and Losa 2022	NAIHA Neurocognitive Test‐Innovative	The NAIHA Neurocognitive Test is a PC‐based cognitive assessment tool that measures orientation, language, memory, attention, and executive function. Participants complete tasks such as word recall, logical puzzles, and visuospatial challenges. Outputs include a total score that reflects cognitive performance across all domains. The tool is used for early detection of cognitive decline, particularly in the elderly population.
Zhang et al. 2023	Hybrid Trail Making Test (TMT)‐Traditional test‐based	The Hybrid Trail Making Test (TMT) is a combination of paper‐based and digital cognitive assessment tools designed to assess executive function and cognitive flexibility. Participants connect numbered and lettered dots in sequence, with the system tracking time and accuracy. Outputs include total task completion time and error rates, which help identify cognitive impairments related to attention and executive functioning. The tool offers a hybrid approach for cognitive testing.
Klil‐Drori et al. 2024	XpressO‐Innovative	The XpressO is a web‐based cognitive testing platform that evaluates memory recall, executive function, and processing time. Participants complete tasks that assess memory retention, problem‐solving, and reaction times. Outputs include memory and executive function scores, as well as detailed data on processing speed. This platform is used for both clinical and remote cognitive testing, providing a comprehensive overview of the participant's cognitive health.
Zhang et al. 2023	Digital Geriatric Complex Figure‐Traditional test‐based	The Digital Geriatric Complex Figure is a PC‐based cognitive assessment tool that evaluates visuospatial abilities and memory. Participants are asked to copy and later recall complex figures on a digital device. The tool tracks the drawing process in detail, including accuracy and time taken to complete the figure. Outputs include completion accuracy scores, helping to identify cognitive impairments related to visuospatial and memory deficits in elderly populations.
Ambrosini et al. 2024	IA Voce‐Innovative	IA Voce is an online platform that provides automatic analysis of free speech. Voice recordings are used to extract high‐level acoustic features at different time scales, including voice periodicity, shimmer‐related features, syllabic patterns, and spectral characteristics. The tool assesses speech, attention, and memory, offering non‐invasive, automatic speech analysis for remote monitoring.
Berron et al. 2024	RDMC (Remote Digital Memory Composite)‐Innovative	The Remote Digital Memory Composite (RDMC) is a mobile app‐based tool that measures episodic memory and long‐term recall. It generates a composite memory performance score (z‐score) based on multiple remote memory tests. Designed for remote cognitive assessment, RDMC offers a comprehensive view of memory function and correlates well with established tools like PACC5.
McMurray et al. 2024	Brainfx SCREEN‐Innovative	The Brainfx SCREEN is a comprehensive cognitive screening tool delivered via an app. It covers seven cognitive domains, including abstract reasoning, constructive ability, prioritizing, numerical problem‐solving, visual–spatial ability, divided attention, and route finding. Participants perform a series of tasks, each assessing these domains, with results provided through activity scores, processing speed, and overall performance.
Zhao et al. 2022	DCS (Digital Cognitive Screening)‐Innovative	The Digital Cognitive Screening (DCS) tool is an app‐based cognitive screener that evaluates memory, orientation, and executive function using voice recognition and conversational AI. Participants complete tasks like a delayed word recall test, orientation questions, and an animal fluency test. The DCS automatically generates a total score ranging from 0 to 12, providing a quick and efficient remote assessment of cognitive health.

### 3.3 Cognitive Domains

#### 3.3.1 Memory

When independent older adults are referred for neuropsychological assessments, it is typically due to concerns regarding memory impairment. These issues can sometimes be due to normal aging processes, but may also indicate a pathological condition caused by physical or psychological factors affecting brain function (Read 1987). Memory complaints represent the most common reason for referral to cognitive disorder clinics, hence making them a valuable starting point for consultations. However, these complaints are often nonspecific and can arise from various causes, thereby requiring further assessment to determine the underlying issue (Kipps and Hodges 2005). Memory assessments, and specifically episodic memory, are central to both traditional neuropsychological tests and innovative digital tools developed to capture various aspects of the domain (Spaan et al. 2003). Among the studies selected, taking into account those with high diagnostic accuracy, traditional tools like the MOCA‐CC (Yu et al. 2015) focus heavily on episodic memory and integrate memory tasks with other functions, such as working memory and orientation, to provide a multidimensional screening of cognitive abilities. On the other hand, innovative tools like the Panoramix Suite (Valladares‐Rodriguez et al. 2018; Valladares‐Rodríguez et al. 2022) explore multiple facets of memory through SG. Panoramix employs a series of engaging tasks (i.e., Episodix, Procedurix, and Semantix) to assess episodic and procedural memory. Episodix examines episodic memory (i.e., the ability to recall past events and experiences) through the gamification of the California Verbal Test (Delis et al. 1987). Procedurix, instead, focuses on the ability to recall steps and procedures involved in performing specific tasks or routines. The procedural memory system is essential for everyday tasks by learning and retrieving routines (Schank and Abelson 2013). A gamified version of the Pursuit Rotor Task was developed to assess procedural memory, motor coordination, and executive functions. In this digital version, subjects track a rotating circle using a mouse. Key variables measured include total playing time, tracking accuracy, response time, and time spent on target across four trials with varying rotor speeds and radii. To assess semantic memory, which involves knowledge of facts and meanings (e.g., recognizing objects or recalling common knowledge), the suite features Semantix. This gamified adaptation of the Pyramids and Palm‐trees test (Howard and Patterson 1992) comprises 52 sets of images, where the subject must match a given stimulus to the correct target out of two options. Performance is evaluated based on time spent playing, number of correct matches, errors, omissions, and total correct responses (Valladares‐Rodriguez et al. 2018). Vulnerability to proactive semantic interference (pSI) is a more sensitive early marker of MCI and a stronger predictor of dementia progression than traditional cognitive tests, such as delayed memory, visual reproduction, Trails B, and category fluency. Additionally, pSI‐sensitive measures strongly correlate to amyloid load in older adults, indicating their potential utility in identifying early AD‐related pathology (D. Loewenstein et al. 2015; D. A. Loewenstein et al. 2007). Thus, the Miami Test of Semantic Interference and Learning (MITSI‐L) (Curiel et al. 2016) measures, through a brief, computerized paired‐associate test, the strength of memory binding of semantically related word pairs and introduces a pSI condition requiring participants to make different associations between semantically similar targets. Finally, episodic memory and long‐term recall were also evaluated through a remote digital memory composite (RDMC) score from an unsupervised remote cognitive assessment battery (Berron et al. 2024).

Integrating SG, as in Panoramix, is of particular interest given the capacity to facilitate high participant engagement and enjoyment. More than 93% of participants in the Valladares‐Rodriguez study found these games enjoyable and engaging. Another example of an innovative tool that assesses memory is the VSM test (Zygouris et al. 2017), evaluating memory within a more ecologically valid setting. In this VR‐based test, participants must remember and retrieve items from a virtual store, testing their ability to retain and recall spatial and object information. This mimics everyday challenges and provides a more practical evaluation of memory, particularly for older adults who may struggle with everyday tasks due to cognitive decline.

Working memory (i.e., the ability to hold and manipulate information in the present) is another component often tested in traditional and digital assessments. CogEvo (Ichii et al. 2020) integrates working memory assessments by requiring participants to retain and process information for short durations.

#### 3.3.2 Executive Function

Executive function encompasses higher‐level cognitive processes such as planning, problem‐solving, and the ability to adapt behavior based on feedback. Traditional tools like the dCDT (Müller et al. 2017) are designed to evaluate various components of executive function. In the dCDT, participants are asked to draw a clock face from memory, requiring them to plan, organize, and execute a series of actions in the correct sequence. Paterson et al. (2022) and Ye et al. (2022) include tasks like working memory recall and task‐switching challenges that assess cognitive flexibility. McMurray et al. (2024) evaluate prioritizing and route‐finding through the Brainfx SCREEN, testing the ability to organize, plan, and adapt to changing tasks. The VSM test (Zygouris et al. 2015, 2017) also tests executive function, as participants must plan and navigate through a virtual environment, making decisions about purchases and how to efficiently navigate the virtual space.

#### 3.3.3 Language

Language is less frequently tested than memory or attention but remains a critical cognitive domain, particularly for diagnosing conditions like Primary Progressive Aphasia. In many digital assessments, language is evaluated through a variety of tasks. Tools like the MOCA‐CC (Yu et al. 2015) assess the ability to identify and name objects shown on a screen, evaluate word retrieval skills, and possibly reveal deficits in verbal fluency, often an early sign of conditions like aphasia or dementia. Verbal fluency is another commonly tested area, explored by asking to list words belonging to a specific category, such as animals, within a limited time. This task provides insight into cognitive deficits related to language, often linked to dysfunctions in the frontal or temporal brain regions. Yu et al. (2015) integrated language tasks such as word recall and verbal fluency into the MOCA‐CC, with strong diagnostic outcomes. Verbal memory and word recall are also assessed by digital innovative tools. BrainCheck (Ye et al. 2022) incorporates real‐time processing of verbal stimuli, evaluating not only linguistic recall but also the processing speed and accuracy to respond to verbal information. This dynamic approach provides a deeper understanding of cognitive health and language processing. Ambrosini et al. (2024) introduce an innovative approach using the IA Vocetool, which applies automatic speech analysis to assess cognitive decline. This tool analyzes speech features (e.g., shimmer, syllabic structure, and voice periodicity), allowing for a noninvasive, real‐time assessment of language‐related cognitive changes. The analysis of these vocal biomarkers provides insights into the quality of speech production and underlying cognitive function, thereby facilitating the detection of subtle signs of linguistic decline that might not be captured by traditional tests. This type of real‐time processing enables the evaluation of both language fluency and speech pattern changes, which can be early indicators of neurodegenerative conditions.

#### 3.3.4 Visuospatial Abilities

Visuospatial abilities refer to the brain's ability to process and interpret visual information about where objects are in space. These abilities are critical for navigation, understanding spatial relationships, and performing tasks like driving or using tools. Visuospatial abilities are also assessed by asking participants to complete spatially complex tasks within a digital format (Robens et al. 2019).

The VSM test (Eraslan Boz et al. 2020) is particularly notable for its examination of spatial navigation. Participants must navigate through a virtual store, find items on a list, and remember their locations, closely mirroring real‐world navigation challenges. Regarding adaptations of traditional pen‐and‐paper tests, the dCDT (Müller et al. 2017) also tests visuospatial skills by requiring participants to accurately place numbers and clock hands on a drawn clock face.

#### 3.3.5 Multidomain

Most of the reviewed tools assess multiple cognitive domains simultaneously, providing a more holistic and accurate picture of an individual's cognitive abilities. One of the most accurate and immersive multidomain tools is the VSM (Eraslan Boz et al. 2020; Zygouris et al. 2015, 2017), which uses VR to assess memory, attention, executive function, and spatial navigation. The VSM's real‐world applicability and ability to engage participants in practical tasks provide high diagnostic accuracy, particularly in detecting early cognitive decline. Other traditional tools, like the MOCA‐CC (Yu et al. 2015), also show strong diagnostic reliability in assessing memory, attention, and executive function. Though less immersive than VR‐based tools, the computerized format of MOCA‐CC allows for consistent application and comparison with normative data, enhancing its accuracy in identifying MCI. Similarly, the eSAGE (Scharre et al. 2017) provides a robust digital alternative to traditional paper‐based exams by assessing multiple domains such as memory, language, and attention with good accuracy due to its standardized format. SG‐based tools like the Panoramix Suite (Valladares‐Rodriguez et al. 2018) have also demonstrated high participant engagement and accuracy, particularly in assessing episodic, procedural, and semantic memory. By gamifying the cognitive testing process, Panoramix maintains high levels of user engagement, which correlates with more reliable test results. Tools like the dCDT (Müller et al. 2017) and BrainCheck Battery (Ye et al. 2022) also assess multiple domains, with dCDT focusing on visuospatial and executive function, and BrainCheck covering memory, attention, and processing speed. These offer detailed task‐specific outputs, increasing the precision and reliability of their assessments. Lastly, tools like the CogEvo (Ichii et al. 2020), which measures memory, attention, and executive function using tasks such as reaction times and spatial puzzles, provide highly accurate results by combining real‐time performance metrics with cognitive analysis.

### 3.4 Correlations With Traditional Neuropsychological Test

Many of these innovative tools have demonstrated strong correlations with well‐established neuropsychological measures, confirming their validity and reliability in cognitive assessment. Memória et al. (2014) found a high correlation with the MoCA (*r* = 0.76, *p* < 0.001) (Nasreddine et al. 2005), indicating that the CANS‐MCI tool effectively aligns with traditional cognitive assessments. Similarly, Yu et al. (2015) reported a strong correlation with MoCA (*r* = 0.93, *p* < 0.001) for their computerized version of the test, suggesting high reliability for early detection of cognitive decline. Zygouris et al. (2015) noted a moderate correlation between MMSE (Copeland et al. 2002) and task duration (*r* = −0.209, *p* < 0.05) in their VSM tool, supporting its use in assessing memory and executive function. Other tools, such as the (Valladares‐Rodriguez et al. 2018) Panoramix Suite, showed a positive correlation with the Rey Auditory Verbal Learning Test (RAVLT) (Schmidt 1996) for episodic memory (*r* = 0.68, *p* < 0.01). The (Berron et al. 2024) study found a high correlation with PACC5 (*r* = 0.62) (Papp et al. 2017) for the RDMC tool, demonstrating its accuracy in measuring episodic memory. Additionally, Zhang et al. (2023) reported a strong correlation with traditional Trail Making Test (TMT) scores (Giovagnoli et al. 1996), indicating an association between the hybrid version and traditional executive function and cognitive flexibility measurement.

### 3.5 Diagnostic Accuracy, Participant Satisfaction, and Usability

The diagnostic accuracy of digital cognitive assessment tools varies across different cognitive domains, as reflected by the studies reviewed (Table 1). Based on their diagnostic performance, the most accurate cognitive tests include Yu et al. (2015), Zhao et al. (2022), and Zygouris et al. 2017. The MOCA‐CC (Montreal Cognitive Assessment—Chinese Version) (Yu et al. 2015) demonstrated the highest accuracy, with an AUC of 0.97, sensitivity of 96%, and specificity of 87%, making it particularly effective for early detection of MCI. This computerized tool evaluates attention, executive function, memory, and language, providing comprehensive cognitive screening. Similarly, Zhao et al. (2022) reported an AUC of 0.95, a sensitivity of 90%, and a specificity of 92% for the Digital Cognitive Screening (DCS) tool. Using voice recognition and conversational AI, DCS assesses memory, orientation, and executive function, offering a noninvasive, fully automated method for cognitive screening, making it suitable for remote assessments. The VSM also provided high accuracy, with a sensitivity of 94% and a specificity of 89% (Zygouris et al. 2015, 2017). This VR‐based tool assesses memory, executive function, and spatial navigation by simulating real‐world tasks in a virtual shopping environment to enhance its ecological validity for measuring real‐life cognitive abilities. These tools are characterized by their high diagnostic accuracy, user engagement, and innovative formats, whether through VR, voice interaction, or comprehensive cognitive tasks, making them highly reliable for detecting cognitive decline. Several cognitive tests demonstrate moderate diagnostic accuracy. (Scharre et al. 2017) with the eSAGE tool showed an AUC of 0.78, with a sensitivity of 62% and a specificity of 81%, making it moderately effective for assessing orientation, language, memory, and executive function through app‐based tasks like word recall and orientation questions. Similarly, Müller et al. (2017) with the dCDT reported an AUC of 0.88, with a sensitivity of 81% and specificity of 72%, assessing attention, visuospatial skills, and executive function through the digital clock‐drawing task. Other tests, like Eraslan Boz et al. (2020) with the VSM, showed a sensitivity of 85% and a specificity of 74%, providing moderately accurate results for memory and spatial navigation in a virtual environment. On the other hand, tests with lower accuracy include Ichii et al. (2020), with the CogEvo, which showed a sensitivity of 78% and specificity of 54%, and (Paterson et al. 2022) with the Brain Health Assessment (BHA), with an AUC of 0.67, sensitivity of 65%, and specificity of 70%. Both tools assess attention, memory, and executive function, but their lower specificity indicates a higher likelihood of false positives, making them less reliable for detecting cognitive decline.

Few studies have explicitly examined participant satisfaction and usability, but those investigating these variables have reported positive findings. For instance, Valladares‐Rodriguez et al. (2018) found that 93.4% of participants found the Panoramix games enjoyable and nonirritating, and IA Voce (Ambrosini et al. 2024) reported high levels of participant satisfaction due to the noninvasive nature of the tool. Similarly Ye et al. (2022) noted a 97.3% satisfaction rate for the BrainCheck Battery, indicating strong user approval of the platform. Berron et al. (2024) highlighted high usability for the RDMC app, with participants experiencing low distraction and maintaining high concentration during remote cognitive assessments. Meanwhile, Zhao et al. (2022) reported a 97.5% completion rate for the DCS tool, showing that participants had no difficulties completing the tasks autonomously.

## 4 Discussion

The review revealed a multifaceted collection of digital tools for cognitive screening, including traditional neuropsychological tests digitized to enhance precision and novel, innovative tools leveraging cutting‐edge technologies like VR, SG, and automated speech analysis. Digitized versions of traditional tools, such as the MOCA‐CC (Yu et al. 2015) and dCDT (Müller et al. 2017), demonstrated strong correlations with their traditional counterparts, preserving their diagnostic validity while benefiting from the advantages of digital platforms (e.g., automatic scoring and enhanced precision).

Innovative tools such as the VSM test (Eraslan Boz et al. 2020; Zygouris et al. 2015, 2017) and the Panoramix suite (Valladares‐Rodriguez et al. 2018) use VR and serious gaming environments to create immersive assessments. These tools offer increased ecological validity by simulating real‐world tasks (Eraslan Boz et al. 2020). Their dynamic nature allows for the simultaneous assessment of multiple cognitive domains, providing a holistic view of an individual's cognitive function. Despite the promise of these new tools, several studies (McCarthy et al. 2024; Öhman et al. 2021) noted challenges related to user compliance and the need for further validation, particularly in non‐Western and older populations.

Two broad categories of digital tools emerged: traditional tests adapted for digital platforms and innovative tools employing novel technologies. Tools like the MOCA‐CC (Yu et al. 2015) and BrainCheck (Ye et al. 2022) preserve the structure of traditional tests but automate scoring and timing to improve accuracy. The shift to digital mediums has generally enhanced the accessibility of these tools, allowing for remote administration via smartphones or computers, thus reducing logistical barriers for patients and clinicians (Pillemer et al. 2016; Schlemmer and Desrichard 2018).

The Panoramix suite is an example of an innovative tool that employs gamification and VR to increase engagement and create a more immersive assessment environment. In the latter, tasks like Episodix and Workix can evaluate memory and executive function in an interactive, game‐like format (Valladares‐Rodriguez et al. 2018). Moreover, tools incorporating speech analysis, like IA Voce (Ambrosini et al. 2024), represent a significant advancement in cognitive assessment by noninvasively analyzing vocal biomarkers linked to cognitive decline.

While these tools are technologically advanced, concerns have been raised about variability in hardware, uncontrolled test environments, and technical barriers faced by older populations (Rogers et al. 2013). Indeed, studies have shown that technical issues (e.g., differences in hardware and operating systems) can affect the consistency of assessments, leading to variable outcomes across different platforms. Moreover, older adults may be less familiar with technology, which could influence their performance on digital tests (Öhman et al. 2021).

The cognitive dimensions considered in the digital tools for early identification of cognitive impairment include memory, executive function, attention, visuospatial abilities, and language, with some tools expanding into implicit cognitive processes like reaction time and speech analysis. Memory, particularly episodic memory, is frequently assessed, being one of the earliest affected domains in MCI and NCDs like AD (Bäckman et al. 2001; Chételat et al. 2006; Perri et al. 2007; Piolino et al. 2002). Digital adaptations of traditional tools, such as the MOCA‐CC (Yu et al. 2015), include memory tasks like delayed recall and word list learning, similar to their paper‐based counterparts. This focus on memory is well supported by prior studies (Spaan et al. 2003), consistently showing that episodic memory decline is a key early marker of cognitive impairment (Petersen et al. 1999). Innovative tools, such as the Panoramix suite (Valladares‐Rodriguez et al. 2018), assess both episodic and procedural memory through SG, offering a dynamic and interactive approach that has shown strong correlations with established memory tests, such as the Rey Auditory Verbal Learning Test (RAVLT) (Schmidt 1996).

Executive function is another critical domain, especially in the early stages of cognitive decline (Baudic et al. 2006; Clark et al. 2012; Clément et al. 2013). Tools like the dCDT (Müller et al. 2017) assess executive function through tasks involving planning, problem‐solving, and the sequencing required to draw a clock. The VSM test (Zygouris et al. 2015) and SG like Workix in the Panoramix suite (Valladares‐Rodriguez et al. 2018) also measure executive function in real‐world contexts, through navigation and task‐switching. This focus on executive function is consistent with earlier research, highlighting its role in differentiating between normal aging and MCI (Fischer et al. 2007).

Attention is commonly evaluated in both traditional and digital tools, often through reaction time and sustained attention tasks. CogEvo (Ichii et al. 2020) assesses attention, measuring response speed and accuracy, while the VSM (Eraslan Boz et al. 2020; Zygouris et al. 2015, 2017) incorporates attention into virtual shopping tasks. Previous studies emphasize the significance of attention deficits in the progression from MCI to dementia, reinforcing the relevance of this domain in cognitive assessments (Das et al. 2007).

Visuospatial abilities, frequently affected in MCI and dementia (Iachini et al. 2009; Mendez et al. 1990), are another domain assessed by traditional and innovative tools. The dCDT (Müller et al. 2017) measures visuospatial function by analyzing how participants draw clock hands, requiring an understanding of spatial relationships. The VSM (Eraslan Boz et al. 2020; Zygouris et al. 2015, 2017) tests visuospatial abilities immersively by asking participants to navigate and collect items in a virtual store.

The study of changes in language function (e.g., connected speech, naming ability, reading, and writing skills) is not as developed. However, it may provide further insight into both diagnostic and intervention approaches to identify and treat individuals suspected to be at risk of developing AD. Language is less frequently tested than other domains but remains important in comprehensive cognitive assessments (Jokel et al. 2019). Tools like the MOCA‐CC (Yu et al. 2015) and BrainCheck (Ye et al. 2022) assess verbal fluency and naming tasks, integrating language tasks with broader cognitive screening. Studies such as Holsinger et al. (2007) have shown that language deficits, particularly in verbal fluency, are linked to cognitive decline and may indicate early‐onset dementia. Beyond these traditional domains, digital tools also enable the assessment of implicit cognitive processes. For example, IA Voce (Ambrosini et al. 2024) uses speech analysis to evaluate cognitive decline through features like voice periodicity and acoustic patterns. Reaction time and voice analysis offer a noninvasive and real‐time method of capturing cognitive changes (Ambrosini et al. 2024). This added dimension, possible through digital technologies, allows for a richer, more comprehensive evaluation of cognitive function, extending beyond what is typically captured by traditional tests. The cognitive dimensions considered in these digital tools align closely with traditional neuropsychological assessments, but digital technologies enhance the ability to assess these domains more dynamically. Focus on memory, executive function, attention, visuospatial skills, and language remains central in early detection efforts, while innovations like SG and speech analysis provide deeper insights into cognitive decline. These findings align with earlier studies that emphasize the importance of these domains in cognitive assessments and support the potential of digital tools to improve the sensitivity and specificity of early‐stage cognitive impairment detection (Wild et al. 2008).

This review revealed varying levels of diagnostic accuracy among digital cognitive tools, with several showing comparable performance to traditional neuropsychological tests. For instance, the MOCA‐CC (Yu et al. 2015), a computerized version of the MoCA, achieved an AUC of 0.97, along with 96% sensitivity and 87% specificity, underscoring its ability to distinguish between healthy individuals and those with MCI. The Panoramix Suite (Valladares‐Rodriguez et al. 2018), featuring SG to assess memory and executive function, showed over 80% sensitivity and specificity.

The VSM test (Zygouris et al. 2015) showed 85% sensitivity and 74% specificity, but when combined with the MMSE, specificity increased to 92% (Eraslan Boz et al. 2020). This highlights the consistent theme in the review as while digital tools offer promising diagnostic capabilities, their performance improves when combined with traditional assessments or with other digital tools (Valladares‐Rodriguez et al. 2018).

A recurring challenge in the reviewed studies is the lower specificity of some tools, leading to false positives. Öhman et al. (2021) noted that many newer digital tools overdiagnose, particularly in older adults with comorbidities. Remote or at‐home testing environments, as highlighted by (Pillemer et al. 2016), can also contribute to inconsistent results due to distractions or technical issues.

While digital tools hold great potential, improving their specificity remains a key priority before they can serve as standalone diagnostic methods. It is evident that, despite the encouraging levels of sensitivity and specificity exhibited by digital screening tools, it is essential to recognize that they are not designed to replace clinical diagnosis. Rather, their purpose is to provide preliminary indications of potential cognitive decline. A recent meta‐analysis (Magno et al. 2024) has pointed out that, despite the encouraging accuracy estimates of digital screening, the overall quality of the tests remains weak due to the risk of bias and methodological heterogeneity, particularly in the selection of participants and the lack of consistent reference standards. These factors may compromise the accuracy of diagnostic performance and increase the risk of false positives or negatives, leading to unfounded reassurances or unnecessary additional investigations. For this reason, the integration of digital tools into clinical practice should be approached with caution and, preferably, as part of a multimodal and progressive assessment pathway. Therefore, it should be tested whether combining different digital assessments with traditional tests can be more effective than using only one singular diagnostic approach, as supported by Eraslan Boz et al. (2020). Future research should focus on refining algorithms, validating these tools across diverse populations, and conducting longitudinal studies to assess their long‐term utility and diagnostic reliability.

Few studies specifically evaluated participant satisfaction, but those that have done so generally reported positive findings. Tools like Panoramix (Valladares‐Rodriguez et al. 2018) and BrainCheck (Ye et al. 2022) received high satisfaction ratings, with users appreciating the noninvasive and engaging nature of the assessments. Gamified tools, in particular, were well‐received, with over 90% of participants rating Panoramix games as enjoyable. These findings align with prior research suggesting that SG can increase engagement, especially in older adults, who might find traditional tests monotonous or stressful (Tong et al. 2017).

## 5 Conclusion

Despite the appeal, user compliance and technological familiarity are significant concerns. Pillemer et al. (2016) and Öhman et al. (2021) raised issues around privacy concerns, attrition rates, and the technological literacy of older adults, which could limit the widespread adoption of digital tools.

Digital cognitive assessments (e.g., SG, VR tools, and automated speech analysis) represent promising advancements in the early detection of cognitive impairment; however, their widespread adoption is hindered by challenges related to diagnostic accuracy, user compliance, and technological variability. Although several tools, such as MOCA‐CC (Yu et al. 2015) and Panoramix (Valladares‐Rodriguez et al. 2018), have shown strong diagnostic potential, significant gaps remain in validation, particularly in diverse and real‐world settings. Moreover, it is required to involve larger and more diverse populations, as most of the current research has been limited to small or geographically restricted groups, affecting the generalizability of the findings. Future studies should prioritize including broader populations, considering variations in education, socioeconomic status, and familiarity with technology (Ganguli et al. 2004). Hence, research should focus on refining these tools, standardizing their use, and validating them across broader populations.

A significant consideration for the future use of digital cognitive tools in intervention studies concerns their test–retest reliability. Although preliminary evidence of acceptable temporal stability has been demonstrated in some digital adaptations of traditional tests (e.g., MOCA‐CC, Yu et al. 2015), many innovative tools (e.g., VR‐based tasks or SG) have yet to be systematically evaluated for this property. This limits their immediate application in the assessment of cognitive improvement outcomes. Consequently, future validation efforts should prioritize test–retest reliability assessment to determine the suitability of these instruments for longitudinal and interventional designs. To ensure advancements in neuropsychological assessments, exploring combined approaches that integrate traditional tests with innovative digital tools is crucial. Evidence suggests that merging these methods can enhance diagnostic accuracy and provide more precise and comprehensive evaluations (Eraslan Boz et al. 2020). New technologies, such as IA Voce (Ambrosini et al. 2024) and VR‐based assessments, present exciting opportunities, yet their efficacy still needs to be validated. To ensure their long‐term utility and diagnostic accuracy, large‐scale longitudinal studies are required (Ambrosini et al. 2024), to help confirm the potential of these tools in clinical practice. Recent advancements in mobile health and wearable technology have further expanded the possibilities for remote cognitive monitoring. Specifically, a substantial ongoing study in the United States investigated the feasibility of utilizing iPhone and Apple Watch data to identify individuals with mild cognitive impairment. Preliminary results demonstrated strong viability, high user adherence, and promising longitudinal validity (Butler et al. 2025). These findings suggest that consumer‐grade wearable devices have the potential to serve as cost‐effective and ecologically sustainable platforms for large‐scale cognitive health monitoring in elderly populations. Therefore, future advancements should focus on developing hybrid models capable of gathering data from various cognitive domains, leading to an extensive understanding of brain functions (Eraslan Boz et al. 2020). Integrating traditional tests with innovative digital approaches may offer the most reliable path forward, ensuring early and accurate identification of neurocognitive disorders.

Moreover, it is essential to address the technological barriers that could hinder the widespread adoption of these tools. Variability in user engagement, technological literacy, and device compatibility are significant concerns. Future research should include usability testing and assess user compliance to ensure these tools can be effectively implemented in clinical and nonclinical settings (Pillemer et al. 2016).

In conclusion, future advancements must prioritize integrating traditional and digital methods, expanding studies to encompass larger and more diverse populations, validating emerging technologies, and identifying and mitigating technological barriers to ensure widespread and effective implementation in real‐world settings.

## Author Contributions

**Ester Cornacchia:** conceptualization (equal), investigation (equal), methodology (equal), project administration (equal), visualization (equal), writing – original draft (equal). **Aurora Bonvino:** conceptualization (equal), investigation (equal), methodology (equal), project administration (equal), resources (equal), validation (equal), visualization (equal), writing – original draft (equal). **Giorgia Francesca Scaramuzzi:** methodology (equal), writing – review and editing (equal). **Daphne Gasparre:** writing – review and editing (equal). **Roberta Simeoli:** conceptualization (equal), writing – review and editing (equal). **Davide Marocco:** conceptualization (equal), project administration (equal), writing – review and editing (equal). **Paolo Taurisano:** conceptualization (equal), project administration (equal), supervision (equal), writing – review and editing (equal).

## Conflicts of Interest

The authors declare no conflicts of interest.

## Related WIREs Articles

Ethical issues when using digital biomarkers and artificial intelligence for the early detection of dementia

## Data Availability

Data sharing is not applicable to this article as no new data were created or analyzed in this study.

## References

[wcs70009-bib-0001] Ambrosini, E. , C. Giangregorio , E. Lomurno , et al. 2024. “Automatic Spontaneous Speech Analysis for the Detection of Cognitive Functional Decline in Older Adults: Multilanguage Cross‐Sectional Study.” JMIR Aging 7: e50537. 38386279 10.2196/50537 PMC11091799

[wcs70009-bib-0002] Bäckman, L. , B. J. Small , and L. Fratiglioni . 2001. “Stability of the Preclinical Episodic Memory Deficit in Alzheimer's Disease.” Brain 124, no. 1: 96–102. 11133790 10.1093/brain/124.1.96

[wcs70009-bib-0071] Alegret, M. , N. Muñoz , N. Roberto , et al. 2020. “A Computerized Version of the Short Form of the Face‐Name Associative Memory Exam (FACEmemory®) for the Early Detection of Alzheimer's Disease.” Alzheimer's Research & Therapy 12, no. 1. 10.1186/s13195-020-00594-6. PMC7077028 32178724

[wcs70009-bib-0003] Battista, P. , C. Salvatore , and I. Castiglioni . 2017. “Optimizing Neuropsychological Assessments for Cognitive, Behavioral, and Functional Impairment Classification: A Machine Learning Study.” Behavioural Neurology 2017: 1–19. 10.1155/2017/1850909. PMC5307249 28255200

[wcs70009-bib-0004] Baudic, S. , G. Dalla Barba , M. C. Thibaudet , A. Smagghe , P. Remy , and L. Traykov . 2006. “Executive Function Deficits in Early Alzheimer's Disease and Their Relations With Episodic Memory.” Archives of Clinical Neuropsychology 21, no. 1: 15–21. 16125364 10.1016/j.acn.2005.07.002

[wcs70009-bib-0005] Berron, D. , W. Glanz , L. Clark , et al. 2024. “A Remote Digital Memory Composite to Detect Cognitive Impairment in Memory Clinic Samples in Unsupervised Settings Using Mobile Devices.” NPJ Digital Medicine 7, no. 1: 79. 38532080 10.1038/s41746-024-00999-9 PMC10965892

[wcs70009-bib-0006] Butler, J. , T. J. Watermeyer , E. Matterson , E. G. Harper , and M. Parra‐Rodriguez . 2024. “The Development and Validation of a Digital Biomarker for Remote Assessment of Alzheimer's Diseases Risk.” DIGITAL HEALTH 10: 20552076241228416. 10.1177/20552076241228416. 38269369 PMC10807338

[wcs70009-bib-0007] Butler, P. M. , J. Yang , R. Brown , et al. 2025. “Smartwatch‐and Smartphone‐Based Remote Assessment of Brain Health and Detection of Mild Cognitive Impairment.” Nature Medicine 31: 829–839. 10.1038/s41591-024-03475-9 PMC11922773 40038507

[wcs70009-bib-0008] Cerasuolo, M. , S. De Marco , R. Nappo , R. Simeoli , and A. Rega . 2024. “The Potential of Virtual Reality to Improve Diagnostic Assessment by Boosting Autism Spectrum Disorder Traits: A Systematic Review.” Advances in Neurodevelopmental Disorders 9: 1–22. 10.1007/s41252-024-00413-1.

[wcs70009-bib-0077] Cheah, W.‐T. , J.‐J. Hwang , S.‐Y. Hong , et al. 2022. “A Digital Screening System for Alzheimer Disease Based on a Neuropsychological Test and a Convolutional Neural Network: System Development and Validation.” JMIR Medical Informatics 10, no. 3: e31106. 10.2196/31106. 35262497 PMC8943541

[wcs70009-bib-0009] Chételat, G. , B. Desgranges , and F. Eustache . 2006. “Brain Profile of Hypometabolism in Early Alzheimer's Disease: Relationships With Cognitive Deficits and Atrophy.” Revue Neurologique 162, no. 10: 945–951. 17028562 10.1016/s0035-3787(06)75104-9

[wcs70009-bib-0010] Clark, L. R. , D. M. Schiehser , G. H. Weissberger , D. P. Salmon , D. C. Delis , and M. W. Bondi . 2012. “Specific Measures of Executive Function Predict Cognitive Decline in Older Adults.” Journal of the International Neuropsychological Society 18, no. 1: 118–127. 22115028 10.1017/S1355617711001524 PMC3314335

[wcs70009-bib-0011] Clément, F. , S. Gauthier , and S. Belleville . 2013. “Executive Functions in Mild Cognitive Impairment: Emergence and Breakdown of Neural Plasticity.” Cortex 49, no. 5: 1268–1279. 22841389 10.1016/j.cortex.2012.06.004

[wcs70009-bib-0012] Copeland, J. R. M. , M. T. Abou‐Saleh , and D. G. Blazer . 2002. Principles and Practice of Geriatric Psychiatry. Wiley. 10.1002/0470846410.

[wcs70009-bib-0013] Curiel, R. E. , E. Crocco , M. Rosado , et al. 2016. “A Brief Computerized Paired Associate Test for the Detection of Mild Cognitive Impairment in Community‐Dwelling Older Adults.” Journal of Alzheimer's Disease 54, no. 2: 793–799. 10.3233/JAD-160370 PMC5610962 27567839

[wcs70009-bib-0014] Das, S. K. , P. Bose , A. Biswas , et al. 2007. “An Epidemiologic Study of Mild Cognitive Impairment in Kolkata, India.” Neurology 68, no. 23: 2019–2026. 10.1212/01.wnl.0000264424.76759.e6. 17548552

[wcs70009-bib-0015] De Anda‐Duran, I. , P. Sunderaraman , E. Searls , et al. 2024. “Comparing Cognitive Tests and Smartphone‐Based Assessment in 2 US Community‐Based Cohorts.” Journal of the American Heart Association 13, no. 2: e032733. 10.1161/JAHA.123.032733. 38226519 PMC10926794

[wcs70009-bib-0016] Delis, D. C. , J. H. Kramer , E. Kaplan , and B. A. Ober . 1987. California Verbal Learning Test, 1st Version, Manual. Psychological Corporation Harcourt Brace Jovanovich.

[wcs70009-bib-0017] Eraslan Boz, H. , H. Limoncu , S. Zygouris , et al. 2020. “A New Tool to Assess Amnestic Mild Cognitive Impairment in Turkish Older Adults: Virtual Supermarket (VSM).” Aging, Neuropsychology, and Cognition 27, no. 5: 639–653. 10.1080/13825585.2019.1663146. 31482749

[wcs70009-bib-0080] Eurostat . 2023. “Towards Digital Decade Targets for Europe—Statistics Explained.” https://ec.europa.eu/eurostat/statistics‐explained/index.php?title=Towards_Digital_Decade_targets_for_Europe.

[wcs70009-bib-0018] Fischer, P. , S. Jungwirth , S. Zehetmayer , et al. 2007. “Conversion From Subtypes of Mild Cognitive Impairment to Alzheimer Dementia.” Neurology 68, no. 4: 288–291. 10.1212/01.wnl.0000252358.03285.9d. 17242334

[wcs70009-bib-0019] Folstein, M. F. , S. E. Folstein , and P. R. McHugh . 1975. ““Mini‐Mental State”: A Practical Method for Grading the Cognitive State of Patients for the Clinician.” Journal of Psychiatric Research 12, no. 3: 189–198. 1202204 10.1016/0022-3956(75)90026-6

[wcs70009-bib-0072] Fung, A. W.‐T. , and L. C. W. Lam . 2018. “Validation of a Computerized Hong Kong – Vigilance and Memory Test (HK‐VMT) to Detect Early Cognitive Impairment in Healthy Older Adults.” Aging & Mental Health 24, no. 1: 186–192. 10.1080/13607863.2018.1523878. 30270640

[wcs70009-bib-0020] Ganguli, M. , H. H. Dodge , C. Shen , and S. T. DeKosky . 2004. “Mild Cognitive Impairment, Amnestic Type: An Epidemiologic Study.” Neurology 63, no. 1: 115–121. 10.1212/01.WNL.0000132523.27540.81. 15249620

[wcs70009-bib-0021] Giovagnoli, A. R. , M. Del Pesce , S. Mascheroni , M. Simoncelli , M. Laiacona , and E. Capitani . 1996. “Trail Making Test: Normative Values From 287 Normal Adult Controls.” Italian Journal of Neurological Sciences 17, no. 4: 305–309. 10.1007/BF01997792. 8915764

[wcs70009-bib-0022] Holsinger, T. , J. Deveau , M. Boustani , and J. W. Williams . 2007. “Does This Patient Have Dementia?” JAMA 297, no. 21: 2391–2404. 17551132 10.1001/jama.297.21.2391

[wcs70009-bib-0023] Howard, D. , and K. E. Patterson . 1992. “The Pyramids and Palm Trees Test.” https://eprints.ncl.ac.uk/666.

[wcs70009-bib-0024] Iachini, T. , A. Iavarone , V. P. Senese , F. Ruotolo , and G. Ruggiero . 2009. “Visuospatial Memory in Healthy Elderly, AD and MCI: A Review.” Current Aging Science 2, no. 1: 43–59. 20021398 10.2174/1874609810902010043

[wcs70009-bib-0025] Ichii, S. , T. Nakamura , T. Kawarabayashi , et al. 2020. “CogEvo, a Cognitive Function Balancer, Is a Sensitive and Easy Psychiatric Test Battery for Age‐Related Cognitive Decline.” Geriatrics & Gerontology International 20, no. 3: 248–255. 10.1111/ggi.13847. 31851431 PMC7187406

[wcs70009-bib-0026] Jokel, R. , B. Seixas Lima , A. Fernandez , and K. J. Murphy . 2019. “Language in Amnestic Mild Cognitive Impairment and Dementia of Alzheimer's Type: Quantitatively or Qualitatively Different?” Dementia and Geriatric Cognitive Disorders Extra 9, no. 1: 136–151.

[wcs70009-bib-0075] Karapapas, C. , and C. Goumopoulos . 2021. “Mild Cognitive Impairment Detection Using Machine Learning Models Trained on Data Collected from Serious Games.” Applied Sciences 11, no. 17: 8184. 10.3390/app11178184.

[wcs70009-bib-0027] Kipps, C. M. , and J. R. Hodges . 2005. “Cognitive Assessment for Clinicians.” Journal of Neurology, Neurosurgery & Psychiatry 76, no. suppl 1: i22–i30. 15718218 10.1136/jnnp.2004.059758 PMC1765683

[wcs70009-bib-0079] Klil‐Drori, S. , K. C. Bodenstein , S. Sun , et al. 2024. “Montreal Cognitive Assessment (MoCA) XpressO: Validation of a Digital Self‐Administered Cognitive Prescreening Tool.” Journal of the American Geriatrics Society 72, no. 8: 2516–2522. 10.1111/jgs.18902. 38558263 PMC11323193

[wcs70009-bib-0076] Konstantinidis, E. I. , P. D. Bamidis , A. Billis , P. Kartsidis , D. Petsani , and S. G. Papageorgiou . 2021. “Physical Training In‐Game Metrics for Cognitive Assessment: Evidence from Extended Trials with the Fitforall Exergaming Platform.” Sensors 21, no. 17: 5756. 10.3390/s21175756. 34502647 PMC8434168

[wcs70009-bib-0028] Livingston, G. , J. Huntley , A. Sommerlad , et al. 2020. “Dementia Prevention, Intervention, and Care: 2020 Report of the Lancet Commission.” Lancet 396, no. 10248: 413–446. 32738937 10.1016/S0140-6736(20)30367-6 PMC7392084

[wcs70009-bib-0029] Loewenstein, D. , R. E. Curiel , M. Greig‐Custo , et al. 2015. “O1‐03‐05: The Relationship Between a Novel Test of Semantic Interference (LASSI‐L) and Global and Regional Accumulation of Amyloid in the Brains of Community‐Dwelling Elders.” Alzheimer's & Dementia 11, no. 7S_Part_3: P131. 10.1016/j.jalz.2015.07.043.

[wcs70009-bib-0030] Loewenstein, D. A. , A. Acevedo , J. Agron , and R. Duara . 2007. “Stability of Neurocognitive Impairment in Different Subtypes of Mild Cognitive Impairment.” Dementia and Geriatric Cognitive Disorders 23, no. 2: 82–86. 17124415 10.1159/000097304

[wcs70009-bib-0031] Luongo, M. , R. Simeoli , D. Marocco , N. Milano , and M. Ponticorvo . 2024. “Enhancing Early Autism Diagnosis Through Machine Learning: Exploring Raw Motion Data for Classification.” PLoS One 19, no. 4: e0302238. 38648209 10.1371/journal.pone.0302238 PMC11034672

[wcs70009-bib-0032] Magno, M. , A. I. Martins , J. Pais , A. G. Silva , and N. P. Rocha . 2024. “Diagnostic Accuracy of Digital Solutions for Screening for Cognitive Impairment: A Systematic Review and Meta‐Analysis.” Applied Sciences 14, no. 6: 2640.

[wcs70009-bib-0033] McCarthy, B. , J. K. Sabharwal , and S. Chawla . 2024. “Old Age or Cognitive Decline? Examining the Usability of a Mobile Health App for Older Australians.” Informatics for Health and Social Care 49, no. 1: 83–97. 10.1080/17538157.2024.2332691. 38529731

[wcs70009-bib-0034] McMurray, J. , A. Levy , W. Pang , and P. Holyoke . 2024. “Psychometric Evaluation of a Tablet‐Based Tool to Detect Mild Cognitive Impairment in Older Adults: Mixed Methods Study.” Journal of Medical Internet Research 26: e56883. 38640480 10.2196/56883 PMC11069099

[wcs70009-bib-0035] Memória, C. M. , M. S. Yassuda , E. Y. Nakano , and O. V. Forlenza . 2014. “Contributions of the Computer‐Administered Neuropsychological Screen for Mild Cognitive Impairment (CANS‐MCI) for the Diagnosis of MCI in Brazil.” International Psychogeriatrics 26, no. 9: 1483–1491. 24806666 10.1017/S1041610214000726

[wcs70009-bib-0036] Mendez, M. F. , M. A. Mendez , R. Martin , K. A. Smyth , and P. J. Whitehouse . 1990. “Complex Visual Disturbances in Alzheimer's Disease.” Neurology 40, no. 3_part_1: 439. 10.1212/WNL.40.3_Part_1.439. 2314585

[wcs70009-bib-0037] Müller, S. , O. Preische , P. Heymann , U. Elbing , and C. Laske . 2017. “Increased Diagnostic Accuracy of Digital vs. Conventional Clock Drawing Test for Discrimination of Patients in the Early Course of Alzheimer's Disease From Cognitively Healthy Individuals.” Frontiers in Aging Neuroscience 9: 101. 28443019 10.3389/fnagi.2017.00101 PMC5386968

[wcs70009-bib-0038] Nasreddine, Z. S. , N. A. Phillips , V. Bédirian , et al. 2005. “The Montreal Cognitive Assessment, MoCA: A Brief Screening Tool for Mild Cognitive Impairment.” Journal of the American Geriatrics Society 53, no. 4: 695–699. 10.1111/j.1532-5415.2005.53221.x. 15817019

[wcs70009-bib-0039] Öhman, F. , J. Hassenstab , D. Berron , M. Schöll , and K. V. Papp . 2021. “Current Advances in Digital Cognitive Assessment for Preclinical Alzheimer's Disease.” Alzheimer's & Dementia: Diagnosis, Assessment & Disease Monitoring 13, no. 1: e12217. 10.1002/dad2.12217. PMC8290833 34295959

[wcs70009-bib-0078] Oliva, I. , and J. Losa . 2022. “Validation of the Computerized Cognitive Assessment Test: NNCT.” International Journal of Environmental Research and Public Health 19, no. 17: 10495. 10.3390/ijerph191710495. 36078210 PMC9518179

[wcs70009-bib-0040] Papp, K. V. , D. M. Rentz , I. Orlovsky , R. A. Sperling , and E. C. Mormino . 2017. “Optimizing the Preclinical Alzheimer's Cognitive Composite With Semantic Processing: The PACC5.” Alzheimer's & Dementia: Translational Research & Clinical Interventions 3, no. 4: 668–677. 10.1016/j.trci.2017.10.004 PMC5726754 29264389

[wcs70009-bib-0041] Paterson, T. S. , B. Sivajohan , S. Gardner , et al. 2022. “Accuracy of a Self‐Administered Online Cognitive Assessment in Detecting Amnestic Mild Cognitive Impairment.” Journals of Gerontology, Series B: Psychological Sciences and Social Sciences 77, no. 2: 341–350. 34333629 10.1093/geronb/gbab097 PMC8824689

[wcs70009-bib-0042] Perri, R. , L. Serra , G. A. Carlesimo , and C. Caltagirone . 2007. “Amnestic Mild Cognitive Impairment: Difference of Memory Profile in Subjects Who Converted or Did Not Convert to Alzheimer's Disease.” Neuropsychology 21, no. 5: 549–558. 17784803 10.1037/0894-4105.21.5.549

[wcs70009-bib-0043] Petersen, R. C. 2009. “Early Diagnosis of Alzheimer's Disease: Is MCI Too Late?” Current Alzheimer Research 6, no. 4: 324–330. 19689230 10.2174/156720509788929237 PMC3098139

[wcs70009-bib-0044] Petersen, R. C. , G. E. Smith , S. C. Waring , R. J. Ivnik , E. G. Tangalos , and E. Kokmen . 1999. “Mild Cognitive Impairment: Clinical Characterization and Outcome.” Archives of Neurology 56, no. 3: 303–308. 10190820 10.1001/archneur.56.3.303

[wcs70009-bib-0045] Pillemer, F. , R. A. Price , S. Paone , et al. 2016. “Direct Release of Test Results to Patients Increases Patient Engagement and Utilization of Care.” PLoS One 11, no. 6: e0154743. 27337092 10.1371/journal.pone.0154743 PMC4919031

[wcs70009-bib-0046] Piolino, P. , B. Desgranges , K. Benali , and F. Eustache . 2002. “Episodic and Semantic Remote Autobiographical Memory in Ageing.” Memory 10, no. 4: 239–257. 10.1080/09658210143000353. 12097209

[wcs70009-bib-0047] Read, D. E. 1987. “Neuropsychological Assessment of Memory in the Elderly.” Canadian Journal of Experimental Psychology 41: 158–174. 10.1037/h0084358 3502894

[wcs70009-bib-0048] Robens, S. , P. Heymann , R. Gienger , et al. 2019. “The Digital Tree Drawing Test for Screening of Early Dementia: An Explorative Study Comparing Healthy Controls, Patients With Mild Cognitive Impairment, and Patients With Early Dementia of the Alzheimer Type.” Journal of Alzheimer's Disease 68, no. 4: 1561–1574. 10.3233/JAD-181029 30909229

[wcs70009-bib-0073] Rodríguez‐Salgado, A. M. , J. J. Llibre‐Guerra , E. Tsoy , et al. 2021. “A Brief Digital Cognitive Assessment for Detection of Cognitive Impairment in Cuban Older Adults.” Journal of Alzheimer's Disease 79, no. 1: 85–94. 10.3233/jad-200985. PMC8216130 33216033

[wcs70009-bib-0049] Rogers, L. J. , G. Vallortigara , and R. J. Andrew . 2013. Divided Brains: The Biology and Behaviour of Brain Asymmetries. Cambridge University Press. https://books.google.com/books?hl=it&lr=&id=lbchAwAAQBAJ&oi=fnd&pg=PR7&dq=‐Rogers,+L.+J.,+Vallortigara,+G.,+%26+Andrew,+R.+J.+(2013).+Divided+brains:+the+biology+and+behaviour+of+brain+asymmetries.+Cambridge+University+Press.&ots=t4W2D9fqVr&sig=1JmGTAjS8oWVNt‐29r‐P334Gi04.

[wcs70009-bib-0050] Sabbagh, M. N. , M. Boada , and S. Borson . 2020. “Early Detection of Mild Cognitive Impairment (MCI) in an At‐Home Setting.” Journal of Prevention of Alzheimer's Disease 7: 171–178. 10.14283/jpad.2020.22. 32463070

[wcs70009-bib-0051] Sabbagh, M. N. , M. Boada , S. Borson , et al. 2020. “Early Detection of Mild Cognitive Impairment (MCI) in Primary Care.” Journal of Prevention of Alzheimer's Disease 7: 165–170. 10.14283/jpad.2020.21. 32463069

[wcs70009-bib-0052] Schank, R. C. , and R. P. Abelson . 2013. Scripts, Plans, Goals, and Understanding: An Inquiry Into Human Knowledge Structures. Psychology Press. https://www.taylorfrancis.com/books/mono/10.4324/9780203781036/scripts‐plans‐goals‐understanding‐roger‐schank‐robert‐abelson.

[wcs70009-bib-0053] Scharre, D. W. , S. I. Chang , H. N. Nagaraja , N. E. Vrettos , and R. A. Bornstein . 2017. “Digitally Translated Self‐Administered Gerocognitive Examination (eSAGE): Relationship With Its Validated Paper Version, Neuropsychological Evaluations, and Clinical Assessments.” Alzheimer's Research & Therapy 9, no. 1: 44. 10.1186/s13195-017-0269-3. PMC5488440 28655351

[wcs70009-bib-0054] Schlemmer, M. , and O. Desrichard . 2018. “Is Medical Environment Detrimental to Memory? A Test of A White Coat Effect on Older People's Memory Performance.” Clinical Gerontologist 41, no. 1: 77–81. 10.1080/07317115.2017.1307891. 28406393

[wcs70009-bib-0055] Schmidt, M. 1996. Rey Auditory Verbal Learning Test. Western Psychological Services.

[wcs70009-bib-0056] Simeoli, R. , A. Rega , M. Cerasuolo , R. Nappo , and D. Marocco . 2024. “Using Machine Learning for Motion Analysis to Early Detect Autism Spectrum Disorder: A Systematic Review.” Review Journal of Autism and Developmental Disorders, 1–20. 10.1007/s40489-024-00435-4.

[wcs70009-bib-0057] Spaan, P. E. J. , J. G. W. Raaijmakers , and C. Jonker . 2003. “Alzheimer's Disease Versus Normal Ageing: A Review of the Efficiency of Clinical and Experimental Memory Measures.” Journal of Clinical and Experimental Neuropsychology 25, no. 2: 216–233. 10.1076/jcen.25.2.216.13638. 12754679

[wcs70009-bib-0058] Taghavi, M. F. , F. Ghorbani , and M. Delrobaei . 2024. “Development of an Augmented‐Reality‐Based Serious Game: A Cognitive Assessment Study.” IEEE Transactions on Cognitive and Developmental Systems 16, no. 3: 1087–1094. 10.1109/TCDS.2023.3329807.

[wcs70009-bib-0059] Tong, T. , J. H. Chan , and M. Chignell . 2017. “Proceedings of the 26th International Conference on World Wide Web Companion ‐ WWW ‘17 Companion.” In Serious Games for Dementia, 1111–1115. ACM Press. 10.1145/3041021.3054930.

[wcs70009-bib-0060] Tsoi, K. K. , J. Y. Chan , H. W. Hirai , S. Y. Wong , and T. C. Kwok . 2015. “Cognitive Tests to Detect Dementia: A Systematic Review and Meta‐Analysis.” JAMA Internal Medicine 175, no. 9: 1450–1458. 26052687 10.1001/jamainternmed.2015.2152

[wcs70009-bib-0070] van der Hoek, M. D. , A. Nieuwenhuizen , J. Keijer , and J. W. Ashford . 2019. “The MemTrax Test Compared to the Montreal Cognitive Assessment Estimation of Mild Cognitive Impairment.” Journal of Alzheimer's Disease 67, no. 3: 1045–1054. 10.3233/jad-181003. PMC6398548 30776011

[wcs70009-bib-0061] Valladares‐Rodríguez, S. , M. J. Fernández‐Iglesias , L. E. Anido‐Rifón , and M. Pacheco‐Lorenzo . 2022. “Evaluation of the Predictive Ability and User Acceptance of Panoramix 2.0, an AI‐Based E‐Health Tool for the Detection of Cognitive Impairment.” Electronics 11, no. 21: 3424. 10.3390/electronics11213424.

[wcs70009-bib-0062] Valladares‐Rodriguez, S. , R. Pérez‐Rodriguez , J. M. Fernandez‐Iglesias , L. Anido‐Rifón , D. Facal , and C. Rivas‐Costa . 2018. “Learning to Detect Cognitive Impairment Through Digital Games and Machine Learning Techniques: A Preliminary Study.” Methods of Information in Medicine 57, no. 4: 197–207. 10.3414/ME17-02-0011. 30248709

[wcs70009-bib-0063] Wild, K. , D. Howieson , F. Webbe , A. Seelye , and J. Kaye . 2008. “Status of Computerized Cognitive Testing in Aging: A Systematic Review.” Alzheimer's & Dementia 4, no. 6: 428–437. 10.1016/j.jalz.2008.07.003. PMC2645803 19012868

[wcs70009-bib-0074] Yan, M. , H. Yin , Q. Meng , et al. 2021. “A Virtual Supermarket Program for the Screening of Mild Cognitive Impairment in Older Adults: Diagnostic Accuracy Study.” JMIR Serious Games 9, no. 4: e30919. 10.2196/30919. 34870610 PMC8686451

[wcs70009-bib-0064] Ye, S. , K. Sun , D. Huynh , et al. 2022. “A Computerized Cognitive Test Battery for Detection of Dementia and Mild Cognitive Impairment: Instrument Validation Study.” JMIR Aging 5, no. 2: e36825. 10.2196/36825. 35436212 PMC9055476

[wcs70009-bib-0065] Yu, K. , S. Zhang , Q. Wang , et al. 2015. “Development of a Computerized Tool for the Chinese Version of the Montreal Cognitive Assessment for Screening Mild Cognitive Impairment.” International Psychogeriatrics 27, no. 2: 213–219. 10.1017/S1041610214002269. 25362894

[wcs70009-bib-0066] Zhang, W. , X. Zheng , Z. Tang , et al. 2023. “Combination of Paper and Electronic Trail Making Tests for Automatic Analysis of Cognitive Impairment: Development and Validation Study.” Journal of Medical Internet Research 25: e42637. 10.2196/42637. 37294606 PMC10337362

[wcs70009-bib-0067] Zhao, X. , R. Hu , H. Wen , et al. 2022. “A Voice Recognition‐Based Digital Cognitive Screener for Dementia Detection in the Community: Development and Validation Study.” Frontiers in Psychiatry 13: 899729. 10.3389/fpsyt.2022.899729. 35935417 PMC9354045

[wcs70009-bib-0068] Zygouris, S. , D. Giakoumis , K. Votis , et al. 2015. “Can a Virtual Reality Cognitive Training Application Fulfill a Dual Role? Using the Virtual Supermarket Cognitive Training Application as a Screening Tool for Mild Cognitive Impairment.” Journal of Alzheimer's Disease 44, no. 4: 1333–1347. 10.3233/JAD-141260. 25428251

[wcs70009-bib-0069] Zygouris, S. , K. Ntovas , D. Giakoumis , et al. 2017. “A Preliminary Study on the Feasibility of Using a Virtual Reality Cognitive Training Application for Remote Detection of Mild Cognitive Impairment.” Journal of Alzheimer's Disease 56, no. 2: 619–627. 10.3233/JAD-160518. 28035922

